# Relating Biopsychosocial Meanings to Semi-Quantitative CRP Readings Among Primary Healthcare Users: Trends from a Cross-Sectional Study Analysis

**DOI:** 10.3390/jcm14124236

**Published:** 2025-06-14

**Authors:** Panagiotis Volkos, Manolis Linardakis, Emmanouil K. Symvoulakis

**Affiliations:** Clinic of Social and Family Medicine, School of Medicine, University of Crete, 71003 Heraklion, Greece; linman@med.uoc.gr (M.L.); esymvoulakis@uoc.gr (E.K.S.)

**Keywords:** C-reactive protein, dopamine, BMI, loneliness, personal sociability

## Abstract

**Background/Objectives:** C-reactive protein (CRP) has been acknowledged to be associated with depression, loneliness, and stress, as well as physical health conditions. The aim of this study was to explore possible associations between CRP semi-quantitative readings and demographic, social, behavioral, and biomedical indices. **Methods**: Group sampling took place between May and July 2023, and from the 120 non-obese participants initially registered in the study, a random selection of n = 80 was performed for blood sampling in order to measure plasma semi-quantitative CRP (higher levels or ≥10 mg/L and lower or <10) and plasma dopamine. Blood sample collection took place between December 2023 and June 2024. Personal sociability, subjective loneliness, and perceived stress were assessed using relevant scales. Hierarchical multiple logistic regression analysis was also performed. **Results**: A unit increase in Body Mass Index (BMI) was related to higher odds for greater semi-quantitative CRP levels (OR = 1.26, *p* = 0.033) while for each unit increase in the Personal Sociability and Connections scale (PeSCs), the odds for higher CRP levels decreased (OR = 0.91, *p* = 0.025). Lower age (OR = 0.90, *p* = 0.009) and higher number of prescribed medications per day (OR = 4.21, *p* = 0.049) also showed significant associations with high semi-quantitative CRP levels. Plasma dopamine and other scale scores used did not show significant associations (*p* > 0.05), despite some interesting descriptive trends. **Conclusions**: The presented results suggest that age, BMI, number of prescribed medications per day, sociability, and CRP readings are constellated in everyday consultations.

## 1. Introduction

C-reactive protein (CRP), using a brief description, is a liver-produced acute-phase protein [1] produced mainly in liver hepatocytes, which is classified as an inflammatory marker [2]. Usually, CRP levels seem to increase in response to infections, injuries, or inflammation [1,2]. Increased CRP levels are related to various diseases including cardiovascular morbidity, depression, several types of cancers, and even frailty and mortality. The literature suggests that elevated CRP levels are linked to elevated in-hospital mortality for both cardiovascular disease (CVD) and non-CVD deaths [1]. All-cause mortality has also been correlated with high levels of high-sensitivity CRP [3]. Additionally, higher CRP levels were associated with the prediction of 12 cancer types, according to the results of a cohort study [4]; meanwhile, in another cohort, higher CRP levels were found among the frail group (compared to healthy and in-between groups) [5].

Furthermore, increased inflammation may be observed as a response to several everyday social stressors [6]. Daily discrimination among black individuals and socioeconomic status trajectories among white individuals have been reported to be correlated with higher CRP levels [7], highlighting the importance of several non-biological variables in the study of inflammation and CRP. Two meta-analyses have reported direct associations between depression and inflammation, as measured by CRP, in both adolescents and adults [8,9].

According to a study, loneliness—as a subjective feeling of social isolation—has been correlated with CRP in a sample of middle-aged participants [10], while another study reported comparable associations among elderly individuals [11]. Additionally, social isolation—measured according to living arrangement (living alone or with others), the availability of at least a couple of people to talk to about ‘important issues’, participation to religious services, and engaging in group activities—has been shown to have a positive association with CRP [12]. Fewer social ties has been previously associated with greater CRP levels in men above 60 years old [13].

Concerning mental health and inflammation, it has been reported that some stress and anxiety disorders could be associated with increased levels of CRP [14], which shows the relationship between psychological conditions and inflammation. Moreover, psychosocial stress likewise impacts CRP levels, further burdening people’s health [15] and exacerbating psychosocial and medical conditions. In addition, CRP levels were negatively correlated with keeping existent friends and positively with making new friends [16], indicating that possible stress deriving from the energy spent to create new social relationships could also affect physical health.

An interesting study in rhesus monkeys reported that CRP, dopamine, and dietary habits that lead to obesity show an intriguing connection [17], demonstrating the complexity of living organisms. Furthermore, it has been found that endorphins and dopamine influence human sociality, as measured by three social domains—namely, disposition, romantic relationships, and social network [18]—while chronic social isolation was related to increased dopamine D2 receptor levels in mice [19]. Given that both CRP and dopamine seem to be associated with similar variables, such as psychosocial status, it seems appropriate to study both markers at the same time.

To summarize, inflammatory markers like CRP may be interrelated with social, mental, emotional, and psychological issues, indicating the complex and multidimensional relationships of human biology. Given the importance of CRP biomarkers in disease development, it may be considered crucial to explore all the aspects that could influence inflammatory biomarkers. Therefore, in order to understand and deal with health issues, multidisciplinary research is needed for explanation of those ‘complex’ phenomena. According to the above and current knowledge, there is a clear scarcity of studies about sociability—describing the tendency of people to create social relationships—and inflammatory or other laboratory markers, especially among humans. However, sociality has been related to fewer absolute white blood cells (WBCs), indicating a decrease in inflammation markers [20]. Additionally, social support and high sensitivity CRP were reversely correlated in children and adolescents [21].

The primary aim of the study was to find possible associations between semi-quantitative (SQ) CRP levels and personal sociability, loneliness, or perceived stress. Secondary objectives aimed to explore the relationships between CRP SQ readings and demographic, social, behavioral, and health indices.

## 2. Materials and Methods

The present cross-sectional study was conducted at the 4th Topiki Monada Ygeias (TOMY) (Local Health Unit) of Heraklion, Crete, Greece. It is a primary healthcare (PHC) unit based in an urban area, and the study sample was pooled from the registered adults. In order to be included, participants had to be between 40 and 75 years old, able to communicate in the Greek language (including reading, writing, and understanding the language), and with a Body Mass Index (BMI) ≤ 29.9 kg/m^2^. Furthermore, the study’s exclusion criteria included severe head trauma or major psychiatric disorder and pregnancy or breastfeeding. Study sampling was performed between May and July 2023, and involved 120 participants [22]. From the 120 participants initially included in the study, a random selection of 80 was performed for the purpose of the current research. Patients were invited to voluntarily provide consent and to agree to offer medical information. Available routine laboratory measurements, taken six months before or upon study initiation, were also used for cross-analysis. Appointments for blood sampling were scheduled in order to extract plasma or serum and prospectively measure hormones and SQ CRP for the needs of a wider research project which is beyond the aims of the current report. Early statistical analysis to detect eventual significant differences between the initially included 120 participant group and the 80-participant sub-group, currently involved for sampling, were performed in order to verify that the two groups maintained their composition and harmonization of features.

### 2.1. Data Collection

Collected information included gender, age, height, weight, education status, family status, number of prescribed medications per day, smoking status, presence of a chronic disease, and hours of nocturnal sleep. A range of available routine biochemical parameters, as previously described, were grouped for the needs of study data entry. These included WBC (K/μL), serum glucose (mg/dL), triglycerides (mg/dL), total cholesterol (mg/dL), high- and non-high-density lipoprotein (HDL) (mg/dL), and low-density lipoprotein cholesterol (LDL-C) (mg/dL). Blood sample collection for plasma semi-quantitative CRP (mg/L) and dopamine (ng/mL) was used for the current cross-sectional design. Dopamine and other hormones have been measured for the needs of a wider research project, which is beyond the aims of the current report. Blood samples were collected between December 2023 and June 2024. All participants were informed about their test results.

### 2.2. Personal Trust and Connection Scale (PeSCs)

The PeSCs scale was initially developed and validated using a Greek sample; it consists of 10 short questions and measures the tendency of a person to shape social connections in daily life [23]. In this context, a 5-point Likert scale response was adopted, as previously suggested (1: never, 2: a few times, 3: some times, 4: most of the time, 5: all the time) [24]. Upon summing up the answers, a composite score was produced, ranging from 10 to 50. The score was categorized as low–moderate (10–39) and high (40–50), where high corresponds to “most of the time” and “all the time”. Cronbach’s alpha was currently estimated at 0.857.

### 2.3. University of California, Los Angeles (UCLA) Loneliness Scale

The UCLA Loneliness Scale (Version 3) was employed in order to determine levels of subjective feelings of loneliness [25]. The version in Greek has been validated by Pikea and colleagues (2016) [26]. The scale includes 20 items, of which 11 are expressed in a negative manner and 9 in a positive manner. A Likert scale of 4 points was used for the responses, ranging from 1 (never) to 4 (always). The final score was assessed after reversing items 1, 5, 6, 9, 10, 15, 16, 19, and 20. Then, all items were added up in order to form a total score between 20 and 80. A high-scale score was related to heightened subjective loneliness feelings. A total score <28 indicated no or low sense of loneliness, while 28 to 43 indicated a moderate sense, and >43 a high sense of loneliness [27,28]. Its reliability at the initial study sample was assessed with a Cronbach’s α value of 0.880 [22].

### 2.4. Perceived Stress Scale (PSS)-14

For evaluating perceived stress, the PSS (14-item version) scale was used [29], which has been validated for the Greek population [30]. Questions were answered with the use of a 5-point Likert scale (0 = never to 4 = very often). Increased scale scores demonstrate higher perceived scores, and items were reversed where necessary [30]. The scale’s reliability was assessed with a Cronbach’s α of 0.786.

### 2.5. Blood Sample Collection

In order to measure plasma dopamine and SQ CRP values, blood samples were collected from the antecubital vein between 08:00 a.m. and 08:30 a.m., in a sitting position. The following instructions were given to the participants: overnight fasting, no alcohol consumption the day before measurement, and smoking abstinence in the morning before blood collection. Blood samples were collected between December 2023 and June 2024.

### 2.6. Dopamine

Total blood from all enrolled participants (n = 80) was collected in 5 mL EDTA treated tubes and centrifuged at 1600× *g* for plasma isolation. All plasma samples were further spun down at 10,000× *g* for 10 min at 4 °C to eliminate the platelet effect. Samples were stored according to the manufacturer’s instructions.

Plasma dopamine was assessed in each sample using a commercial ELISA kit from Abcam (Cambridge, UK) (Cat. No. ab285238), in accordance with the instructions given by the manufacturer. Hormone concentrations were determined after fitting optical density values in standard curves obtained for dopamine.

### 2.7. CRP

CRP was assessed using a CRP Semi-Quantitative Rapid Test Cassette from RightSign (Hanghzou, China) (REF CCRP-C43), in accordance with the instructions provided by the manufacturer. Total blood from all enrolled participants was collected in 5 mL EDTA treated tubes and centrifuged at 1600× *g* for plasma isolation. According to the manufacturer, the thresholds for detecting CRP values are as follows: less than 10 mg/L is a negative result while 10–40 mg/L, 40–80 mg/L, and ≥80 mg/L are positive results.

Information from the manufacturer summarizes that a semi-quantitative CRP test is a non-time-consuming chromatographic immunoassay for detecting a range of increased CRP levels. Due to its low-cost, quick, and simple implementation, it may be a useful tool for the early detection of elevated CRP levels before proceeding in quantitative CRP procedures. Physicians in remote areas or in healthcare units with limited resources—as in primary healthcare, in some cases—could use semi-quantitative CRP tests for the rapid evaluation of a person’s health situation before they are able to perform or refer to more specialized care and practices.

### 2.8. Ethics

The study was approved by the Ethics and Deontology Committee of the University of Crete (ref. no. 166/11.11.2022; approval date: 11 November 2022) and by the 7th Health Regional Authority of Crete (ref. no. 6460; approval date: 8 February 2023). The study was conducted according to the Declaration of Helsinki. All study participants gave written informed consent.

### 2.9. Statistical Analysis

Data analysis was performed with the IBM SPSS software (IBM Corp. Released 2019, IBM SPSS Statistics for Windows, v.25.0, Armonk, NY, USA). Frequency distributions of the basic characteristics and biochemical markers for the 80 participants were calculated. Blom’s method (Q-Q plot) was used to assess the normality of data. Chi-square (χ^2^) and Mann–Whitney tests were utilized to compare characteristics, scales scores, and biochemical markers in terms of SQ CRP levels (higher levels or ≥10 mg/L and lower or <10mg/L). Moreover, based on hierarchical multiple logistic regression analysis, two models were developed to assess the higher levels of SQ CRP in relation to the characteristics, sense of loneliness, and sociability scale of the participants. The acceptable level of significance was set at 5%.

## 3. Results

In the current sample, 78.8% of participants were women and the mean age was 59.4 years (±8.9) (Table 1). Concerning their education level, 31.3% reported tertiary level of education, and 65.0% of participants were married. The mean BMI was estimated at 26 kg/m^2^, with 65.0% being overweight. Mean plasma dopamine was 7.65 ng/mL (±0.39), and 25 participants (31.3%) indicated SQ CRP levels higher or equal to 10 mg/L.

Furthermore, the sample was divided in two groups, those with ≥10 mg/L SQ CRP levels (n = 25) and those with <10 mg/L SQ CRP levels (n = 55) (Table 2), which was the threshold for “positive” SQ CRP according to the manufacturer. It was estimated that those with elevated SQ CRP levels (≥10 mg/L) had lower mean PeSCs scale levels in relation to their counterparts (<10 mg/L) (33.9 vs. 37.5, respectively, *p* = 0.031).

Additionally, according to Table 3, 40.4% of the overweight participants had SQ CRP levels ≥10 mg/L compared to 14.3% of those with normal weight (*p* = 0.011).

As demonstrated in Table 4, each additional year of increasing age corresponds to decreased odds for elevated SQ CRP levels (OR = 0.90, *p* = 0.007), while the odds for elevated SQ CRP levels increased among those who use five or more prescribed medications per day (OR = 4.80, *p* = 0.024). Additionally, in the second, more complete model, age (OR = 0.90, *p* = 0.009) and number of prescribed medications per day (OR = 4.21, *p* = 0.049) continue to provide significant associations in terms of high SQ CRP levels. Additionally, a unit increase in BMI corresponds to increased odds for higher SQ CRP levels (OR = 1.26, *p* = 0.033), while for each unit increase in PeSCs, the odds for higher SQ CRP levels decrease (OR = 0.91, *p* = 0.025).

## 4. Discussion

The presented results suggest that age, BMI, number of prescribed medications per day, and levels of Personal Sociability and Connections are associated with the levels of SQ CRP. An increase per year in terms of age indicates lower odds for increased SQ CRP levels, while those who receive five or more prescribed medications per day showed higher odds. Moreover, each increase in BMI was significantly correlated with higher odds for elevated SQ CRP, while each unit increase on the PeSCs scale was associated with lower odds for higher SQ CRP levels.

Descriptively, among those participants with SQ CRP ≥ 10 mg/L, a lower mean of WBC count, less hours of sleep, a higher serum glucose level, a higher total cholesterol/HDL ratio, a higher LDL/HDL ratio (>2), and a higher non-HDL (mean > 130 mg/dL) were found compared to those with CRP < 10 mg/L, as shown in Table 2. Although these comparisons did not reach statistical significance, probably due to a limited sample size, a ‘grayer’ profile towards cardiovascular risk appears as a trend. It seems that silent systemic inflammation may occur if we consider that participants with SQ CRP ≥ 10 mg/L were slightly younger but with a high probability of taking more medicines for chronic conditions. In a previous study, we assessed that younger age is related to more multiple behavioral risk factors and that a sense of ‘invulnerability’ may be related to neglecting risk [31]. According to the literature, an increased WBC count is related to higher CVD risk [32], and CRP levels between 3 and 10 μg/mL indicate high risk for CVD while CRP > 10 μg/mL (or 10 mg/L) requires further investigation [33]. The association of CRP with personal sociability highlights the importance of future multidisciplinary efforts that include social, emotional, and behavioral aspects and laboratory markers or disease outcomes.

The findings regarding the relationship between BMI and CRP are consistent with other studies, despite most of them usually including samples with a wider BMI range than the present study sample [34,35]. However, it has been reported that the threshold for positive associations between BMI and CRP could be set at BMI > 24.3 kg/m^2^ [35]. The present results could suggest that BMI is correlated with CRP levels even in non-obese individuals, and the role of metabolic disorders should be further investigated. Additionally, the positive relationship trend that was observed among prescribed medication intake and markers of inflammation has been reported in other studies as well, even though CRP was not included [36], while polypharmacy (receiving more than four medications per day) and elevated interleukin (IL)-6 levels (another inflammatory marker) were related to frailty in older individuals [37], revealing the meaning of studying those variables when assessing health profiles in a more comprehensive and thorough manner.

In relation to age, our results are in contrast with other research initiatives reporting that age and CRP are positively associated. More specifically, the study of Wyczalkowska-Tomasik and colleagues (2015) showed that individuals between 60 and 70 years old were found to have higher CRP levels than their younger counterparts; however, their levels were within the normal ranges [38]. Another study highlighted that people aged above 45 years old showed greater levels of CRP than those less than 45 years old [39]. The present negative association between SQ CRP and age may be explained by the fact that all participants were registered frequent primary care attendees who probably had routine visits for prescriptions. Furthermore, it is interesting that despite the absence of a significantly different metabolic profile, BMI shows a significant association with SQ CRP levels in both univariate and multivariate analyses, a finding that should be separately discussed from co-existing variables.

A study among rhesus macaques reported that greater sociality levels were correlated with reduced numbers of WBCs, which is one of the main elements regarding inflammation and may designate decreased inflammation levels [20]. According to an interesting review [40], social support as well as social integration were found to be correlated with decreased levels of inflammatory markers, including CRP, indicating similar results to the present survey. Social isolation during childhood was related to elevated levels of inflammation markers, again including CRP, later in life [41]. To the authors’ knowledge, the relationships between CRP and sociability have not been widely studied as a triangular research hypothesis of clinical, psychometric, and laboratory input. If social support or isolation are the epiphenomena of individual and systemic influences, we wonder to what extent the tendency to be social contributes to a possible risk buffering mechanism.

Moreover, loneliness levels and perceived stress appeared to be slightly higher among those with SQ CRP ≥10 mg/L. However, those associations were not significant. Despite that, previous research reported similar associations, where increased loneliness was related to higher CRP levels in both middle aged [10] and older participants [42]. Furthermore, it should not be ignored that loneliness is positively associated with LDL/HDL atherosclerotic index [22] and negatively associated with HDL [43]. Additionally, the positive relationships between social isolation and loneliness [44] should not be overlooked. In the literature, perceived stress was positively related to CRP in a longitudinal study with mid-adolescent to young adults [45]. Another study, which included a middle-aged sample, again concluded that perceived stress was higher among those with increased levels of inflammatory markers, CRP included [46]. In fact, the authors suggested that stress may be associated with a higher risk for metabolic disease [46]. However, it is worth recognizing that in this context PSS-14 was used. Its retrospective design, assessing stress over the previous four weeks, may be a limitation. Since the lipid profile and metabolism can be influenced by behavioral or emotional factors, further research is needed to decode messaging connections between them. Furthermore, a connection between emotional, behavioral, social, and psychological factors with various physical health outcomes can be observed, indicating the need for holistic scientific approaches.

Last but not least, mean plasma dopamine levels were found to be slightly lower in the participants with SQ CRP ≥10 mg/L; however, those correlations were also not significant. To the authors’ knowledge, research that studies the direct relationship between CRP and dopamine is limited. A recent study revealed a negative association between dopamine D2-receptor availability and levels of inflammation, measured using the CRP-associated DNA methylation scores [47]. Interestingly, CRP levels and the response to dopaminergic therapies in individuals with depression were reported to be related [48], a finding that indicates that these are complex pathways deserving more research to build on. Further research regarding the role of neuropeptides should be undertaken. Additionally, a larger pool of participants, quantitative CRP measurements, and longitudinal methodology could further cast light on these complex observations.

Adding personal sociability to the knowledge toolkit, creating tools to measure hidden related modulators, or using psychosocial interventions along with traditional medicine may help to better manage attendees within a PHC setting. Multidisciplinary approaches are required to implement interventions and create PHC environments suitable for hosting such teams and concepts. Furthermore, these findings could be taken into account when policy makers design and implement message campaigns where, for instance, empowering psychosocial resilience and the way of communication may be helpful for dealing with non-communicable risk factors.

### Strengths and Limitations

One of the study’s strengths refers to the use of a validated tool that measures personal sociability, while most studies in the field of inflammatory markers explore associations with loneliness or social isolation. In this context, personal sociability appears to have an independent impact as an earlier temporal determinant of active or passive choices. Higher personal sociability was related to lower SQ CRP readings. On the other hand, higher SQ CRP readings among usual PHC attendees, recruited for non-clinical reasons, were related to a more demanding health profile, with increased BMI and more prescribed medications per day and less sociability. Additionally, the sample consisted of non-obese individuals; thus, elevated SQ CRP levels could not be confounded with obesity. Moreover, a significant association emerged between SQ CRP measurements and increased BMI, despite a cut-off ≤29.9 kg/m^2^. SQ CRP was associated only with PeSCs and not with perceived stress, loneliness, or even with neuropeptides, such as dopamine, indicating a specific cross-sectional interaction between those two variables. Furthermore, a limited sample size could not be excluded as a factor that may have affected the lack of associations between CRP and dopamine, loneliness, or perceived stress. However, plasma dopamine cannot offer explanations on central nervous system pathways driven by dopamine-mediating effects.

A meta-analysis reported that social isolation was related to CRP and fibrinogen, while loneliness was related to IL-6 [49]. However, no correlation was found between loneliness and CRP and fibrinogen [49]. According to the authors of this meta-analysis, the terms “social isolation” and “loneliness” should not be used interchangeably as they probably affect inflammatory markers within a differential manner [49]. In addition, another study reported that living alone (as an indication for social isolation) was related to lower changes in cortisol levels during the day and increased CRP levels [50]. Loneliness and IL-6 were positively correlated, while loneliness was not found to mediate the influence of social isolation [50]. The authors also highlighted that loneliness and social isolation should be treated as distinguished variables when attempting to explain their impact on inflammation or endocrine markers [50].

From our multiple regression model analysis, and despite the fact that our study had a limited sample size, age, BMI, polypharmacy, and sociability, but not loneliness, were significantly predictive of the semi-quantitative CRP reading trends. This leads us to rationally state that a pre-clinical impact occurs within the present findings. In a primary care environment, which differs from the general population context, one can easily ‘screen’ for such variables, and if all are detectable, a scenario for an occult systemic inflammatory process might then likely be supported by an abnormal CRP as well. This pre-clinical awareness can prompt decision-making to follow a clinical assessment pathway well before major clinical symptoms occur. Primary care needs evidence, combined with its own practice modality inputs, that can be smoothly tested and applied with the use of metric tool/s and feasibly measurable markers. This pre-clinical utility is prioritized since sociability—as the tendency to be connected—is perceived to be closer to social isolation than loneliness based on the current findings, in terms of its relationship with an inflammatory marker such as semi-quantitative CRP. Semi-quantitative CRP appears to be more easily used in primary care, despite the weaker information that it provides. Furthermore, BMI was found to have a relationship with semi-quantitative CRP readings among overweight individuals, which supports an earlier clinical suspicion of an occult inflammatory process—without waiting to diagnose obesity as an already alarming finding. This study offers a proof-of-concept dimension to relate pre-clinical practice with a decision to clinically assess bio-behavioral risk factors within future interdisciplinary research.

Despite this, the study bares some limitations as well. First of all, causality is difficult to establish due to the study’s cross-sectional design. More specifically, it is not clear whether elevated CRP levels are the consequence of reduced sociability, or whether increased CRP may contribute to lower sociability levels. The sample is not sufficiently able to detect significant associations with systemic metabolic inflammation. Large population samples may offer this opportunity after properly selecting a mixture of biopsychosocial variables. SQ CRP tests did not allow the designation of the specific CRP levels of each participant but instead categorized them into CRP ranges. This led to the choice to analyze CRP as a discrete variable. The SQ CRP method may be less accurate for the determination of precise CRP measurements since it provides categorical input rather than continuous values; nevertheless, it may be used by physicians in cases in which limited resources or referral services are available. For those reasons, it could be a useful tool in PHC, especially when they are placed in remote areas. For similar study objectives and hypotheses, lack of funding is a further limitation to take into consideration. Moreover, the size of the sample is definitely considered limited for generalizing the present findings. However, the study’s aim was not to generalize the results but mainly to use them as a starting point for broader research. Future efforts could investigate possible associations among personal sociability and inflammatory or other laboratory biomarkers with larger samples, a cohort design, and CRP assessment with higher sensitivity. Additionally, the sample consisted of non-obese individuals within a specific age range. It would be interesting to assess the likelihood of our findings without BMI cut-off points and by measuring related cytokines such as IL-6 or IL-8 in order to gain a better understanding.

## 5. Conclusions

According to the results, polypharmacy, somatometric features (such as BMI), age, and psychosocial parameters (such as personal sociability) can offer interesting information when inflammatory markers such as SQ CRP readings are available. The aforementioned findings indicate the importance of multidimensional approaches at the clinical, research, and health promotion levels. In a primary care environment, a healthcare professional can easily ‘screen’ for the aforementioned variables. If they are all detected and if abnormal CRP levels are found, a scenario for an occult systemic inflammatory process might then likely be supported. This pre-clinical screening could lead to further clinical processes and actions of assessment, much before a major clinical alertness might arise. Semi-quantitative CRP, due to its non-time-consuming use and low-cost availability, appears to be more easily used in primary care despite the weaker information that it provides. It should not be used as a substitute for quantitative CRP evaluation, if available, or should be clinically performed. There is a need to focus on multidisciplinarity when trying to holistically address health issues. Dealing with the cure of a symptom or condition is different from curing the person as a complex and unique being. Public health and primary care may also have an important role to play in this matter, since BMI and social connections are both linked to inflammation. The role of systemic inflammation should be investigated in depth, using larger samples, particularly at the intersection where primary care meets public health.

## Figures and Tables

**Table 1:** Basic characteristics and biochemical markers of the 80 participants.

		n	%
Gender	Male/female	17/63	21.3/78.8
Age, years	Mean ± standard deviation (min–max)	59.4 ± 8.9 (40–75)	
Education	Primary school	10	12.5
	Junior high school, high school, technical education	45	56.2
	University/Technological School	24	30.0
	PhD, MSc	1	1.3
Family status	Unmarried, divorced, widow	28	35.0
	Married	52	65.0
Body mass index, kg/m^2^	Mean ± standard deviation	26.0 ± 3.1	
	Normal weight	28	35.0
	Overweight	52	65.0
Plasma dopamine, ng/mL	Mean ± standard deviation	7.65 ± 0.39	
Semi-quantitative C-reactive protein, mg/L	<10	55	68.8
	≥10	25	31.3

**Table 2:** Characteristics, scales scores, and biochemical markers of the 80 study participants, in terms of semi-quantitative C-reactive protein (CRP) levels.

	Semi-Quantitative CRP		
	<10 mg/L (n = 55)	≥10 mg/L (n = 25)	
	Mean ± Standard Deviation		*p*-Value
Age, years	60.4 ± 8.9	56.6 ± 8.6	0.113
Body mass index, kg/m^2^	25.5 ± 3.3	27.0 ± 2.4	0.095
Hours of nocturnal sleep	6.9 ± 1.2	6.5 ± 1.4	0.136
Personal sociability and connections scale (PeSCs) ^a^	37.5 ± 6.1	33.9 ± 8.2	0.031
Perceived stress scale 14 (PSS-14) ^b^	24.3 ± 7.6	25.7 ± 8.4	0.493
UCLA loneliness scale ^c^	40.8 ± 9.8	41.9 ± 8.7	0.663
White blood cells, K/μL	6.99 ± 2.10	6.53 ± 1.44	0.506
Serum glucose, mg/dL	101 ± 18	109 ± 32	0.736
Total cholesterol, mg/dL	185.3 ± 39.7	194.2 ± 37.4	0.309
>200 mg/dL or medication	63.6%	80.0%	0.143
Total cholesterol to HDL ratio	3.21 ± 0.87	3.59 ± 1.29	0.364
Triglycerides to HDL ratio	2.23 ± 1.72	2.24 ± 1.71	0.868
LDL to HDL ratio	1.82 ± 0.66	2.11 ± 0.96	0.337
non-HDL, mg/dL	123.7 ± 33.5	135.6 ± 42.8	0.290
LDL-C, mg/dL	104 ± 31	112 ± 31	0.327
Plasma dopamine, ng/mL	7.66 ± 0.41	7.61 ± 0.35	0.427

^a^ The Personal Sociability and Connections scale consists of 10 questions, each based on a 5-point Likert answer scale (1: never, 2: a few times, 3: some times, 4: most of the time, and 5: all the time). Summing up the answers, a composite score is produced ranging from 10 to 50, thus categorized as low–moderate (10–39) and high (40–50), where high corresponds to most of the time and all the time. ^b^ The Perceived Stress Scale 14 includes 14 questions (items), in a graded, closed-type Likert scale (0: never to 4: very often). Summing up the answers, a composite score is produced with a range of 0 to 56. A higher score (➡56) shows higher stress. ^c^ Range score between 20 and 80 where a higher score demonstrates increased sense of subjective loneliness. Mann–Whitney tests.

**Table 3:** Demographic characteristics and levels of loneliness of the 80 study participants, in terms of semi-quantitative C-reactive protein (CRP) levels.

		Semi-Quantitative CRP		
		<10 mg/L (n = 55)	≥10 mg/L (n = 25)	
		n (%)		*p*-Value
Gender	male	12 (70.6)	5 (29.4)	0.854
	female	43 (68.3)	20 (31.7)	
Body mass index, kg/m^2^	normal	24 (85.7)	4 (14.3)	0.011
	overweight	31 (59.6)	21 (40.4)	
Smoking	no	34 (65.4)	18 (34.6)	0.376
	yes	21 (75.0)	7 (25.0)	
Chronic diseases	no	7 (70.0)	3 (30.0)	0.927
	yes	48 (68.6)	22 (31.4)	
Number of prescribed medications per day	0–4	41 (73.2)	15 (26.8)	0.188
	5+	14 (58.3)	10 (41.7)	
University of California, Los Angeles loneliness scale	low/moderate	32 (66.7)	16 (33.3)	0.622
	high	23 (71.9)	9 (28.1)	
χ^2^ tests				

**Table 4:** Hierarchical multiple logistic regression analysis regarding higher levels of semi-quantitative C-reactive protein (CRP) levels and characteristics, sense of loneliness, and sociability scale of the 80 participants.

	Semi-Quantitative CRP (Higher Levels or ≥10 mg/L Versus Lower or <10)					
	Initial Model			Secondary Model		
Prognostic Factors	Odds Ratio	95% CIs	*p*-Value	Odds Ratio	95% CIs	*p*-Value
Gender (females versus males)	0.99	0.27, 3.65	0.995	1.24	0.32, 4.86	0.755
Age (per year increase)	0.90	0.84, 0.97	0.007	0.90	0.83, 0.97	0.009
Body mass index (per unit increase)	1.21	0.99, 1.47	0.059	1.26	1.02, 1.56	0.033
Prescribed medications per day (5+ versus 0–4)	4.80	1.23, 18.66	0.024	4.21	1.01, 17.61	0.049
University of California, Los Angeles loneliness scale (high versus low, moderate)	-			0.46	0.14, 1.56	0.212
Personal sociability and connections scale (per unit increase)	-			0.91	0.82, 0.99	0.025
R^2^ Nagelkerke	0.224			0.312		

## Data Availability

The data presented in this study are available on request from the corresponding author since they are part of a wider research project.

## Abbreviations

The following abbreviations are used in this manuscript:

BMI	Body Mass Index
CRP	C-Reactive Protein
CVD	Cardiovascular Disease
HDL	High-Density Lipoprotein
IL	Interleukin
LDL-C	Low-Density Lipoprotein Cholesterol
PSS	Perceived Stress Scale
PeSCs	Personal Trust and Connections Scale
PHC	Primary Healthcare
SQ	Semi-Quantitative
TOMY	Topiki Monada Ygeias—Local Health Unit
UCLA	University of California, Los Angeles
WBCs	White Blood Cells
